# (*E*)-Methyl 3-(4-chloro­phen­yl)-2-{2-[(*E*)-(hy­droxy­imino)­meth­yl]phen­oxy­meth­yl}acrylate

**DOI:** 10.1107/S1600536811038372

**Published:** 2011-09-30

**Authors:** K. SakthiMurugesan, E. Govindan, J. Srinivasan, M. Bakthadoss, A. SubbiahPandi

**Affiliations:** a Department of Physics, Presidency College (Autonomous), Chennai 600 005, India; b Department of Organic Chemistry, University of Madras, Guindy Campus, Chennai 600 025, India

## Abstract

In the title compound, C_18_H_16_ClNO_4_, the dihedral angle between the mean planes through the aromatic rings is 83.8 (8)°. The hy­droxy­ethanimine group is essentially coplanar with the ring to which it is attached [O—N—C—C torsion angle = −177.96 (13)°]. The mol­ecules are linked into centrosymmetric *R*_2_^2^(6) dimers *via* O—H⋯N hydrogen bonds. The crystal packing is further stabilized by C—H⋯O inter­actions.

## Related literature

For the biological activity of caffeic acids, see: Hwang *et al.* (2001 ▶); Altug *et al.* (2008 ▶); Ates *et al.* (2006 ▶); Atik *et al.* (2006 ▶); Padinchare *et al.* (2001 ▶). For the use of oxime ligands in coordination chemistry, see: Chaudhuri (2003 ▶). For related structures, see: Wang *et al.* (2011 ▶); Govindan *et al.* (2011 ▶).
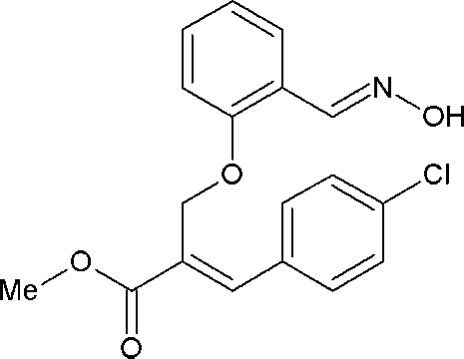


## Experimental

### 

#### Crystal data

C_18_H_16_ClNO_4_
*M* *_r_* = 345.77
Triclinic, 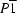

*a* = 8.8860 (4) Å
*b* = 9.3428 (5) Å
*c* = 12.1494 (6) Å
α = 72.289 (3)°
β = 74.319 (2)°
γ = 63.429 (2)°
*V* = 848.63 (7) Å^3^
*Z* = 2
Mo *K*α radiation
μ = 0.25 mm^−1^
*T* = 293 K
0.25 × 0.22 × 0.19 mm

#### Data collection

Bruker APEXII CCD area-detector diffractometer
Absorption correction: multi-scan (*SADABS*; Sheldrick, 1996 ▶) *T*_min_ = 0.978, *T*_max_ = 0.983
22640 measured reflections
6056 independent reflections
4013 reflections with *I* > 2σ(*I*)
*R*_int_ = 0.023

#### Refinement

*R*[*F* ^2^ > 2σ(*F* ^2^)] = 0.051
*wR*(*F* ^2^) = 0.161
*S* = 1.04
6056 reflections
219 parameters
H-atom parameters constrained
Δρ_max_ = 0.39 e Å^−3^
Δρ_min_ = −0.51 e Å^−3^

### 

Data collection: *APEX2* (Bruker, 2004 ▶); cell refinement: *SAINT* (Bruker, 2004 ▶); data reduction: *SAINT*; program(s) used to solve structure: *SHELXS97* (Sheldrick, 2008 ▶); program(s) used to refine structure: *SHELXL97* (Sheldrick, 2008 ▶); molecular graphics: *PLATON* (Spek, 2009 ▶); software used to prepare material for publication: *SHELXL97* and *PLATON*.

## Supplementary Material

Crystal structure: contains datablock(s) global, I. DOI: 10.1107/S1600536811038372/bt5635sup1.cif

Structure factors: contains datablock(s) I. DOI: 10.1107/S1600536811038372/bt5635Isup2.hkl

Supplementary material file. DOI: 10.1107/S1600536811038372/bt5635Isup3.cml

Additional supplementary materials:  crystallographic information; 3D view; checkCIF report

## Figures and Tables

**Table 1:** Hydrogen-bond geometry (Å, °)

*D*—H⋯*A*	*D*—H	H⋯*A*	*D*⋯*A*	*D*—H⋯*A*
O1—H1*A*⋯N1^i^	0.82	2.12	2.8309 (16)	145
C15—H15⋯O3^ii^	0.93	2.38	3.186 (2)	145

Symmetry codes: (i) 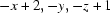; (ii) 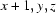.

**Fig. 1.**
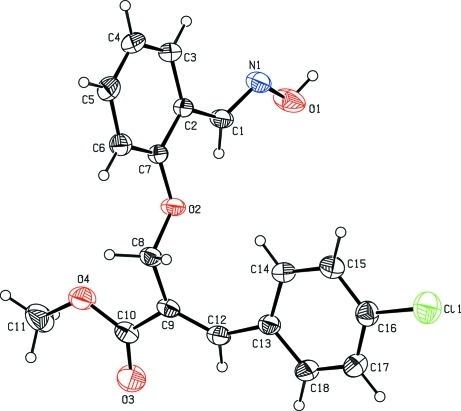
View of the title molecule with the atom labelling scheme. The displacement ellipsoids are drawn at the 30% probability level while the H atoms are shown as small spheres of arbitrary radii.

**Fig. 2.**
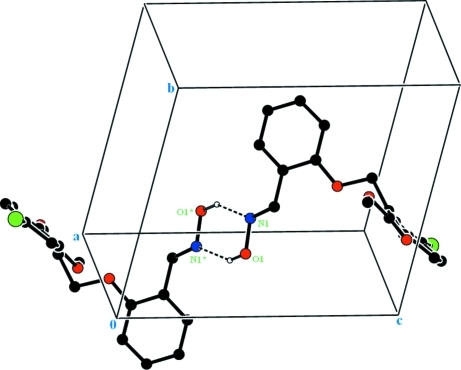
The crystal structure showing the centrosymmetric hydrogen bond motif R22(6). For the sake of clarity, the H atoms not involved in the motif have been omitted. The atoms marked with an asterisk (*) are at the symmetry position (2 - x, -y, 1 - z). The dashed lines indicate the hydrogen bonds.

## supplementary crystallographic information

### Comment

Some naturally occurring caffeic acids and their esters attract much attention in biology and medicine (Hwang *et al.*, 2001; Altug *et al.*, 2008). These compounds show antiviral, antibacterial, vasoactive, antiatherogenic, antiproliferative, antioxidant and antiinflammatory properties (Atik *et al.*, 2006; Padinchare *et al.*, 2001; Ates *et al.*, 2006). Oximes are a classical type of chelating ligands which are widely used in coordination and analytical chemistry (Chaudhuri, 2003). Against this background, and in order to obtain detailed information on molecular conformations in the solid state, an X-ray study of the title compound was carried out.

X-Ray analysis confirms the molecular structure and atom connectivity as illustrated in Fig. 1. The bond lengths and angles in (Fig. 1) agree with those observed in other acrylate derivatives (Wang *et al.*, 2011). The whole molecule is not planar as the dihedral angle between the two phenyl rings is 83.8 (8)°, it shows that both the rings are almost perpendicular to each other. The methoxybutene group connects the two phenyl rings, results in twisting the rings and placed those rings in perpendicular direction. The oxime group having the C═N forming an E configuration. The atom Cl1 is deviated by 0.060 (1)Å from the least squares plane of the C13—C18 ring. The hydroxyethanimine group is essentially coplanar with the benzene ring, the largest deviation from the mean plane being 0.014 (1)Å for the C1 atom.

The enoate group assumes an extended conformation as can be seen from torsion angles C9—C10—O4—C11 [179.3 (1) °] and C12—C9—C10—O4 [-168.9 (1) °]. The hydroxyethanimine group in the molecules are linked into cyclic centrosymmetric dimers *via* O—H···N hydrogen bonds with the motif *R*_2_^2^(6). In addition to van der Waals interaction, the crystal packing is stabilized by C–H..O and O–H···N interactions.

### Experimental

To a stirred solution of (*E*)-methyl 2-((2-formylphenoxy) methyl)-3-(4-chlorophenyl)acrylate (4 mmol) in 10 ml of EtOH/H_2_O mixture (1:1) was added NH_2_OH.HCl (6 mmol) in the presence of 50% NaOH at room temperature. Then the reaction mixture was allowed to stir at room temperature for 1.5 h. After completion of the reaction, solvent was removed and the crude mass was diluted with water (15 ml) and extracted with ethyl acetate (3 *x* 15 ml). The combined organic layer was washed with brine (2 *x* 10 ml) and dried over anhydrous Na_2_SO_4_ and then evaporated under reduced pressure to obtain (*E*)-methyl3- (4-chlorophenyl)-2-((2-((*E*)-(hydroxyimino)methyl)phenoxy)methyl)acrylate as a colourless solid. Single crystals suitable for X-ray diffraction were obtained by slow evaporation of a solution of the title compound in acetone at room temperature.

### Refinement

All H atoms were fixed geometrically and allowed to ride on their parent C atoms, with C—H distances fixed in the range 0.93–0.97 Å with *U*_iso_(H) = 1.5*U*_eq_(C) for methyl H 1.2*U*_eq_(C) for other H atoms.

### Crystal data

**Table:**

C_18_H_16_ClNO_4_	*Z* = 2
*M**_r_* = 345.77	*F*(000) = 360
Triclinic, *P*1	*D*_x_ = 1.353 Mg m^−^^3^
Hall symbol: -P 1	Mo *K*α radiation, λ = 0.71073 Å
*a* = 8.8860 (4) Å	Cell parameters from 6056 reflections
*b* = 9.3428 (5) Å	θ = 2.5–32.5°
*c* = 12.1494 (6) Å	µ = 0.25 mm^−^^1^
α = 72.289 (3)°	*T* = 293 K
β = 74.319 (2)°	Block, white crystalline
γ = 63.429 (2)°	0.25 × 0.22 × 0.19 mm
*V* = 848.63 (7) Å^3^	

### Data collection

**Table:**

Bruker APEXII CCD area-detector diffractometer	6056 independent reflections
Radiation source: fine-focus sealed tube	4013 reflections with *I* > 2σ(*I*)
graphite	*R*_int_ = 0.023
ω and φ scans	θ_max_ = 32.5°, θ_min_ = 2.5°
Absorption correction: multi-scan (*SADABS*; Sheldrick, 1996)	*h* = −13→13
*T*_min_ = 0.978, *T*_max_ = 0.983	*k* = −13→14
22640 measured reflections	*l* = −18→18

### Refinement

**Table:**

Refinement on *F*^2^	Primary atom site location: structure-invariant direct methods
Least-squares matrix: full	Secondary atom site location: difference Fourier map
*R*[*F*^2^ > 2σ(*F*^2^)] = 0.051	Hydrogen site location: inferred from neighbouring sites
*wR*(*F*^2^) = 0.161	H-atom parameters constrained
*S* = 1.04	*w* = 1/[σ^2^(*F*_o_^2^) + (0.0713*P*)^2^ + 0.1584*P*] where *P* = (*F*_o_^2^ + 2*F*_c_^2^)/3
6056 reflections	(Δ/σ)_max_ < 0.001
219 parameters	Δρ_max_ = 0.39 e Å^−^^3^
0 restraints	Δρ_min_ = −0.51 e Å^−^^3^

### Special details

**Table:**

Geometry. All e.s.d.'s (except the e.s.d. in the dihedral angle between two l.s. planes) are estimated using the full covariance matrix. The cell e.s.d.'s are taken into account individually in the estimation of e.s.d.'s in distances, angles and torsion angles; correlations between e.s.d.'s in cell parameters are only used when they are defined by crystal symmetry. An approximate (isotropic) treatment of cell e.s.d.'s is used for estimating e.s.d.'s involving l.s. planes.
Refinement. Refinement of *F*^2^ against ALL reflections. The weighted *R*-factor *wR* and goodness of fit *S* are based on *F*^2^, conventional *R*-factors *R* are based on *F*, with *F* set to zero for negative *F*^2^. The threshold expression of *F*^2^ > σ(*F*^2^) is used only for calculating *R*-factors(gt) *etc*. and is not relevant to the choice of reflections for refinement. *R*-factors based on *F*^2^ are statistically about twice as large as those based on *F*, and *R*- factors based on ALL data will be even larger.

### Fractional atomic coordinates and isotropic or equivalent isotropic displacement parameters (Å^2^)

**Table:**

	*x*	*y*	*z*	*U*_iso_*/*U*_eq_	
C3	0.76040 (18)	0.46783 (19)	0.49605 (13)	0.0517 (3)	
H3	0.8399	0.4114	0.4401	0.062*	
C4	0.7342 (2)	0.6270 (2)	0.48827 (14)	0.0592 (4)	
H4	0.7965	0.6772	0.4281	0.071*	
C5	0.6153 (2)	0.71133 (19)	0.57003 (15)	0.0567 (4)	
H5	0.5972	0.8191	0.5646	0.068*	
C6	0.52179 (19)	0.63806 (17)	0.66070 (13)	0.0487 (3)	
H6	0.4415	0.6963	0.7155	0.058*	
C7	0.54922 (15)	0.47730 (15)	0.66875 (11)	0.0384 (3)	
C2	0.67024 (15)	0.38967 (16)	0.58609 (11)	0.0396 (3)	
C1	0.69450 (17)	0.22183 (17)	0.59516 (12)	0.0457 (3)	
H1	0.6076	0.1872	0.6359	0.055*	
C9	0.25890 (16)	0.35977 (17)	0.91804 (11)	0.0423 (3)	
C12	0.31776 (17)	0.23354 (17)	1.00615 (12)	0.0456 (3)	
H12	0.2466	0.1781	1.0421	0.055*	
C13	0.47403 (17)	0.16776 (16)	1.05564 (12)	0.0441 (3)	
C14	0.62373 (18)	0.18941 (19)	0.99839 (13)	0.0521 (3)	
H14	0.6292	0.2485	0.9215	0.062*	
C15	0.7634 (2)	0.1246 (2)	1.05398 (15)	0.0576 (4)	
H15	0.8618	0.1414	1.0152	0.069*	
C16	0.7569 (2)	0.03523 (19)	1.16672 (14)	0.0548 (3)	
C17	0.6130 (2)	0.0072 (2)	1.22484 (15)	0.0609 (4)	
H17	0.6102	−0.0555	1.3007	0.073*	
C18	0.4742 (2)	0.07297 (19)	1.16918 (14)	0.0548 (4)	
H18	0.3772	0.0539	1.2083	0.066*	
C11	−0.1064 (2)	0.5320 (3)	0.76182 (19)	0.0834 (6)	
H11A	−0.0975	0.4408	0.7358	0.125*	
H11B	−0.1339	0.6279	0.6999	0.125*	
H11C	−0.1945	0.5510	0.8285	0.125*	
C10	0.09091 (18)	0.38980 (19)	0.89357 (13)	0.0497 (3)	
N1	0.83143 (16)	0.12225 (15)	0.54872 (12)	0.0522 (3)	
O1	0.82593 (17)	−0.03007 (15)	0.56443 (13)	0.0761 (4)	
H1A	0.9160	−0.0912	0.5328	0.114*	
O2	0.46629 (12)	0.39236 (12)	0.75426 (8)	0.0464 (2)	
O3	−0.00369 (16)	0.32873 (18)	0.95683 (13)	0.0781 (4)	
O4	0.05387 (15)	0.49645 (18)	0.79367 (11)	0.0693 (3)	
Cl1	0.93049 (7)	−0.04275 (8)	1.23850 (5)	0.0881 (2)	
C8	0.34517 (17)	0.47105 (16)	0.84520 (12)	0.0437 (3)	
H8A	0.2621	0.5754	0.8114	0.052*	
H8B	0.4027	0.4906	0.8930	0.052*	

### Atomic displacement parameters (Å^2^)

**Table:**

	*U*^11^	*U*^22^	*U*^33^	*U*^12^	*U*^13^	*U*^23^
C3	0.0464 (7)	0.0539 (8)	0.0416 (7)	−0.0187 (6)	0.0038 (5)	−0.0038 (6)
C4	0.0572 (8)	0.0555 (9)	0.0522 (8)	−0.0278 (7)	−0.0007 (6)	0.0071 (7)
C5	0.0596 (9)	0.0437 (7)	0.0613 (9)	−0.0233 (7)	−0.0090 (7)	−0.0001 (7)
C6	0.0489 (7)	0.0441 (7)	0.0495 (7)	−0.0176 (6)	−0.0034 (6)	−0.0102 (6)
C7	0.0359 (5)	0.0420 (6)	0.0344 (6)	−0.0160 (5)	−0.0038 (4)	−0.0048 (5)
C2	0.0347 (5)	0.0439 (6)	0.0351 (6)	−0.0142 (5)	−0.0031 (4)	−0.0055 (5)
C1	0.0420 (6)	0.0501 (7)	0.0413 (7)	−0.0204 (6)	0.0056 (5)	−0.0121 (5)
C9	0.0374 (6)	0.0465 (7)	0.0415 (6)	−0.0168 (5)	0.0063 (5)	−0.0182 (5)
C12	0.0432 (6)	0.0468 (7)	0.0458 (7)	−0.0213 (6)	0.0054 (5)	−0.0140 (6)
C13	0.0448 (6)	0.0402 (6)	0.0443 (7)	−0.0177 (5)	0.0023 (5)	−0.0116 (5)
C14	0.0469 (7)	0.0557 (8)	0.0457 (7)	−0.0224 (6)	0.0011 (6)	−0.0046 (6)
C15	0.0478 (8)	0.0616 (9)	0.0604 (9)	−0.0251 (7)	−0.0023 (6)	−0.0090 (7)
C16	0.0557 (8)	0.0506 (8)	0.0561 (9)	−0.0166 (7)	−0.0114 (6)	−0.0133 (7)
C17	0.0661 (10)	0.0559 (9)	0.0491 (8)	−0.0220 (8)	−0.0062 (7)	−0.0015 (7)
C18	0.0549 (8)	0.0492 (8)	0.0524 (8)	−0.0245 (7)	0.0010 (6)	−0.0026 (6)
C11	0.0504 (9)	0.1214 (18)	0.0703 (12)	−0.0190 (10)	−0.0147 (8)	−0.0288 (12)
C10	0.0424 (7)	0.0541 (8)	0.0506 (8)	−0.0168 (6)	0.0015 (5)	−0.0199 (6)
N1	0.0478 (6)	0.0478 (6)	0.0575 (7)	−0.0212 (5)	0.0089 (5)	−0.0180 (5)
O1	0.0708 (8)	0.0564 (7)	0.0973 (10)	−0.0341 (6)	0.0298 (7)	−0.0353 (7)
O2	0.0487 (5)	0.0448 (5)	0.0412 (5)	−0.0218 (4)	0.0115 (4)	−0.0145 (4)
O3	0.0571 (7)	0.0865 (9)	0.0933 (10)	−0.0428 (7)	−0.0108 (6)	−0.0013 (7)
O4	0.0495 (6)	0.1007 (10)	0.0502 (6)	−0.0280 (6)	−0.0061 (5)	−0.0105 (6)
Cl1	0.0749 (3)	0.1059 (4)	0.0811 (4)	−0.0278 (3)	−0.0320 (3)	−0.0120 (3)
C8	0.0416 (6)	0.0430 (6)	0.0425 (7)	−0.0160 (5)	0.0050 (5)	−0.0154 (5)

### Geometric parameters (Å, °)

**Table:**

C3—C4	1.375 (2)	C14—C15	1.377 (2)
C3—C2	1.3911 (18)	C14—H14	0.9300
C3—H3	0.9300	C15—C16	1.372 (2)
C4—C5	1.374 (2)	C15—H15	0.9300
C4—H4	0.9300	C16—C17	1.379 (2)
C5—C6	1.389 (2)	C16—Cl1	1.7336 (17)
C5—H5	0.9300	C17—C18	1.370 (2)
C6—C7	1.3859 (19)	C17—H17	0.9300
C6—H6	0.9300	C18—H18	0.9300
C7—O2	1.3637 (14)	C11—O4	1.444 (2)
C7—C2	1.4015 (17)	C11—H11A	0.9600
C2—C1	1.4570 (19)	C11—H11B	0.9600
C1—N1	1.2649 (17)	C11—H11C	0.9600
C1—H1	0.9300	C10—O3	1.1982 (19)
C9—C12	1.339 (2)	C10—O4	1.327 (2)
C9—C10	1.487 (2)	N1—O1	1.3985 (16)
C9—C8	1.4961 (18)	O1—H1A	0.8200
C12—C13	1.460 (2)	O2—C8	1.4364 (14)
C12—H12	0.9300	C8—H8A	0.9700
C13—C18	1.394 (2)	C8—H8B	0.9700
C13—C14	1.3960 (19)		
			
C4—C3—C2	121.24 (13)	C13—C14—H14	119.5
C4—C3—H3	119.4	C16—C15—C14	119.71 (14)
C2—C3—H3	119.4	C16—C15—H15	120.1
C5—C4—C3	119.48 (13)	C14—C15—H15	120.1
C5—C4—H4	120.3	C15—C16—C17	120.90 (15)
C3—C4—H4	120.3	C15—C16—Cl1	120.00 (13)
C4—C5—C6	121.02 (14)	C17—C16—Cl1	119.11 (13)
C4—C5—H5	119.5	C18—C17—C16	119.01 (15)
C6—C5—H5	119.5	C18—C17—H17	120.5
C7—C6—C5	119.37 (13)	C16—C17—H17	120.5
C7—C6—H6	120.3	C17—C18—C13	121.93 (14)
C5—C6—H6	120.3	C17—C18—H18	119.0
O2—C7—C6	124.59 (12)	C13—C18—H18	119.0
O2—C7—C2	115.15 (11)	O4—C11—H11A	109.5
C6—C7—C2	120.25 (12)	O4—C11—H11B	109.5
C3—C2—C7	118.63 (12)	H11A—C11—H11B	109.5
C3—C2—C1	122.13 (12)	O4—C11—H11C	109.5
C7—C2—C1	119.22 (11)	H11A—C11—H11C	109.5
N1—C1—C2	121.29 (12)	H11B—C11—H11C	109.5
N1—C1—H1	119.4	O3—C10—O4	122.92 (15)
C2—C1—H1	119.4	O3—C10—C9	124.79 (15)
C12—C9—C10	115.36 (12)	O4—C10—C9	112.26 (13)
C12—C9—C8	126.12 (13)	C1—N1—O1	112.01 (12)
C10—C9—C8	118.52 (13)	N1—O1—H1A	109.5
C9—C12—C13	131.27 (12)	C7—O2—C8	118.70 (10)
C9—C12—H12	114.4	C10—O4—C11	116.24 (15)
C13—C12—H12	114.4	O2—C8—C9	107.58 (10)
C18—C13—C14	117.39 (14)	O2—C8—H8A	110.2
C18—C13—C12	116.98 (12)	C9—C8—H8A	110.2
C14—C13—C12	125.62 (13)	O2—C8—H8B	110.2
C15—C14—C13	121.01 (14)	C9—C8—H8B	110.2
C15—C14—H14	119.5	H8A—C8—H8B	108.5
			
C2—C3—C4—C5	−0.8 (3)	C14—C15—C16—C17	1.0 (3)
C3—C4—C5—C6	0.3 (3)	C14—C15—C16—Cl1	−178.50 (13)
C4—C5—C6—C7	0.1 (2)	C15—C16—C17—C18	−1.4 (3)
C5—C6—C7—O2	179.42 (13)	Cl1—C16—C17—C18	178.09 (13)
C5—C6—C7—C2	0.0 (2)	C16—C17—C18—C13	−0.2 (3)
C4—C3—C2—C7	0.9 (2)	C14—C13—C18—C17	2.0 (2)
C4—C3—C2—C1	179.24 (14)	C12—C13—C18—C17	−179.02 (14)
O2—C7—C2—C3	−179.96 (12)	C12—C9—C10—O3	12.6 (2)
C6—C7—C2—C3	−0.48 (19)	C8—C9—C10—O3	−166.71 (15)
O2—C7—C2—C1	1.66 (17)	C12—C9—C10—O4	−168.91 (13)
C6—C7—C2—C1	−178.86 (13)	C8—C9—C10—O4	11.76 (17)
C3—C2—C1—N1	23.8 (2)	C2—C1—N1—O1	−177.96 (13)
C7—C2—C1—N1	−157.87 (14)	C6—C7—O2—C8	−1.97 (19)
C10—C9—C12—C13	−179.66 (13)	C2—C7—O2—C8	177.48 (11)
C8—C9—C12—C13	−0.4 (2)	O3—C10—O4—C11	−2.2 (2)
C9—C12—C13—C18	158.99 (15)	C9—C10—O4—C11	179.32 (14)
C9—C12—C13—C14	−22.1 (2)	C7—O2—C8—C9	173.33 (11)
C18—C13—C14—C15	−2.4 (2)	C12—C9—C8—O2	83.72 (16)
C12—C13—C14—C15	178.71 (14)	C10—C9—C8—O2	−97.03 (13)
C13—C14—C15—C16	1.0 (3)		

### Hydrogen-bond geometry (Å, °)

**Table:**

*D*—H···*A*	*D*—H	H···*A*	*D*···*A*	*D*—H···*A*
O1—H1A···N1^i^	0.82	2.12	2.8309 (16)	145.
C15—H15···O3^ii^	0.93	2.38	3.186 (2)	145.

Symmetry codes: (i) −*x*+2, −*y*, −*z*+1; (ii) *x*+1, *y*, *z*.
